# Prevalence and predictors of burnout among Chinese medical postgraduates: a multicenter cross-sectional study

**DOI:** 10.3389/fpsyg.2026.1888232

**Published:** 2026-10-05

**Authors:** Yi-Le Wu, Na-Na Meng, Meng Wang, Tian-Tai Shao, Yehuan Sun, Wen-Ping Ji

**Affiliations:** 1 Graduate Student Affairs Department, The Second Affiliated Hospital of Anhui Medical University, Hefei, Anhui, China; 2 Development and Reform Department, The Second Affiliated Hospital of Anhui Medical University, Hefei, Anhui, China; 3 Postgraduate and Undergraduate Affairs Department, The Fourth Affiliated Hospital of Anhui Medical University, Chaohu, Anhui, China; 4 Department of Epidemiology and Health Statistics, School of Public Health, Anhui Medical University, Hefei, Anhui, China

**Keywords:** burnout, healthcare, occupational exposure, postgraduates, psychological health

## Abstract

**Background:**

Burnout is a vital concern in healthcare and causes multiple adverse outcomes. This study explored the prevalence of burnout among Chinese medical postgraduates and its associated factors.

**Methods:**

This cross-sectional study was conducted among medical postgraduates at 15 teaching hospitals across eight cities in Anhui Province, China. Structured questionnaires were administered to collect data on sociodemographic characteristics, occupational exposures, occupational safety factors, anxiety symptoms, and burnout. Multivariate linear regression analysis was applied to identify the predictors of burnout.

**Results:**

A total of 1,276 full-time medical postgraduates were included, with a mean age of 26.68 ± 2.65 years and females accounting for 57.29%. Overall, 30.02% of participants reported no burnout, 32.68% mild, 20.22% moderate, and 17.07% severe burnout. In the fully adjusted model, academic postgraduate and strict compliance with standard operating procedures were independently associated with reduced emotional exhaustion; anesthesiology specialty, a history of sharp injury within the past year, concern about occupational exposure, and anxiety symptoms predicted greater emotional exhaustion. Academic postgraduate, pursuing a doctoral degree, intensive care/emergency medicine specialty, strict compliance with standard operating procedures, hospital pre-training safety education, and adequate access to personal protective equipment independently predicted lower depersonalization; surgery and anesthesiology specialties and anxiety symptoms were linked to greater depersonalization. Advanced age and university occupational safety education were independently associated with lower levels of reduced personal accomplishment; anxiety symptoms were significantly correlated with higher levels of reduced personal accomplishment.

**Limitations:**

The cross-sectional design only allows assessment of associations rather than causal relationships; reliance on self-reported questionnaires may introduce response bias and social desirability effects; and the sample recruited solely from Anhui Province limits the generalizability of the findings to other regions.

**Conclusion:**

These findings provide targeted insights for developing effective intervention strategies to alleviate burnout and improve the quality of clinical training.

## 1 Introduction

Burnout is a common psychological syndrome among individuals in high-stress professions, especially within healthcare settings. In academic research, burnout is generally divided into three dimensions, namely emotional exhaustion, depersonalization, and reduced personal accomplishment (Maslach, 1993). These dimensions act synergistically to deplete emotional resources and impair workers’ well-being and occupational performance (Maslach, 1993). Burnout is widely recognized as a major concern in healthcare settings, where healthcare workers routinely face substantial emotional demands (De Hert, 2020). Its adverse impacts extend beyond individual psychological well-being, affecting organizational functioning by increasing turnover rates, reducing work satisfaction, and compromising healthcare quality and patient safety (Menon et al., 2020; Shanafelt et al., 2012).

Healthcare workers are particularly susceptible to burnout owing to long working hours, intense emotional labor, and frequent exposure to patient suffering (Sun et al., 2025). Accumulating evidence demonstrates that physicians and nurses bear a substantially higher risk of burnout compared with other professional populations, with global prevalence estimates ranging from 30% to more than 60% (Alhassan et al., 2025). Epidemiological data reveal a high prevalence of burnout among Chinese healthcare workers, particularly within hospitals with heavy patient loads and inadequate medical resources (Zhang et al., 2021). Medical students constitute an understudied vulnerable group facing distinctive stressors, including intensive academic workloads, early clinical involvement, and uncertain career trajectories, which elevate their burnout risk substantially (Shan et al., 2025; Zhang et al., 2024). Therefore, mitigating burnout among medical students protects their mental health, preserves clinical quality, and promotes healthcare workforce sustainability.

While substantial evidence has addressed burnout, existing research predominantly targets in-service healthcare professionals. Less attention has been paid to burnout and its correlates among Chinese medical postgraduates within local medical education and clinical contexts. Prior evidence suggests that individual demographics, occupational and emotion stress, specialty types, and work environments collectively contribute to burnout outcomes among healthcare workers (Dubale et al., 2019; West et al., 2018). Meanwhile, research targeting medical students has indicated that psychological stress and inadequate social support also serve as vital drivers of burnout occurrence (Li et al., 2026). Moreover, occupational exposures are highly prevalent and non-negligible among healthcare students across various majors (Hambridge, 2023; Huang et al., 2022; Xu et al., 2022). Notably, these occupational adverse events have been documented to be closely associated with burnout in healthcare workers (Ercömert et al., 2025). Nevertheless, the combined effects and interactions of these factors on burnout in medical postgraduates remain under-investigated.

The purpose of this study is to comprehensively investigate the prevalence of burnout among Chinese medical postgraduates, and further explore its associations with demographic factors, occupational exposure, occupational safety support, and psychological stress. By addressing this research gap, this study enriches burnout-related literature and provides practical mitigation recommendations to improve medical education quality and protect psychological well-being among future healthcare professionals.

## 2 Methods

### 2.1 Study setting and participants

This cross-sectional study was conducted in March 2023 among medical postgraduates receiving standardized clinical training at 15 teaching hospitals distributed across eight cities in Anhui Province, China, including Hefei, Fuyang, Huainan, Bengbu, Lu’an, Wuhu, Suzhou, and Anqing. A convenience sampling strategy was utilized, and participants were enrolled in accordance with the following inclusion criteria: (1) full-time medical postgraduate students include professional degree and academic degree postgraduates; (2) aged 18 years or older; (3) second- or third-year postgraduates undergoing specialized clinical training. The study was conducted in accordance with the Declaration of Helsinki. Ethical approval for this study was obtained from the Research Ethics Committees of the Second Affiliated Hospital of Anhui Medical University (YJ-YX2019-033). Prior to data collection, participants were provided with information regarding the voluntary nature, purpose, and detailed procedures, and written informed consent was obtained from all eligible respondents. The study methodology is illustrated in Figure 1.

**Figure 1:**
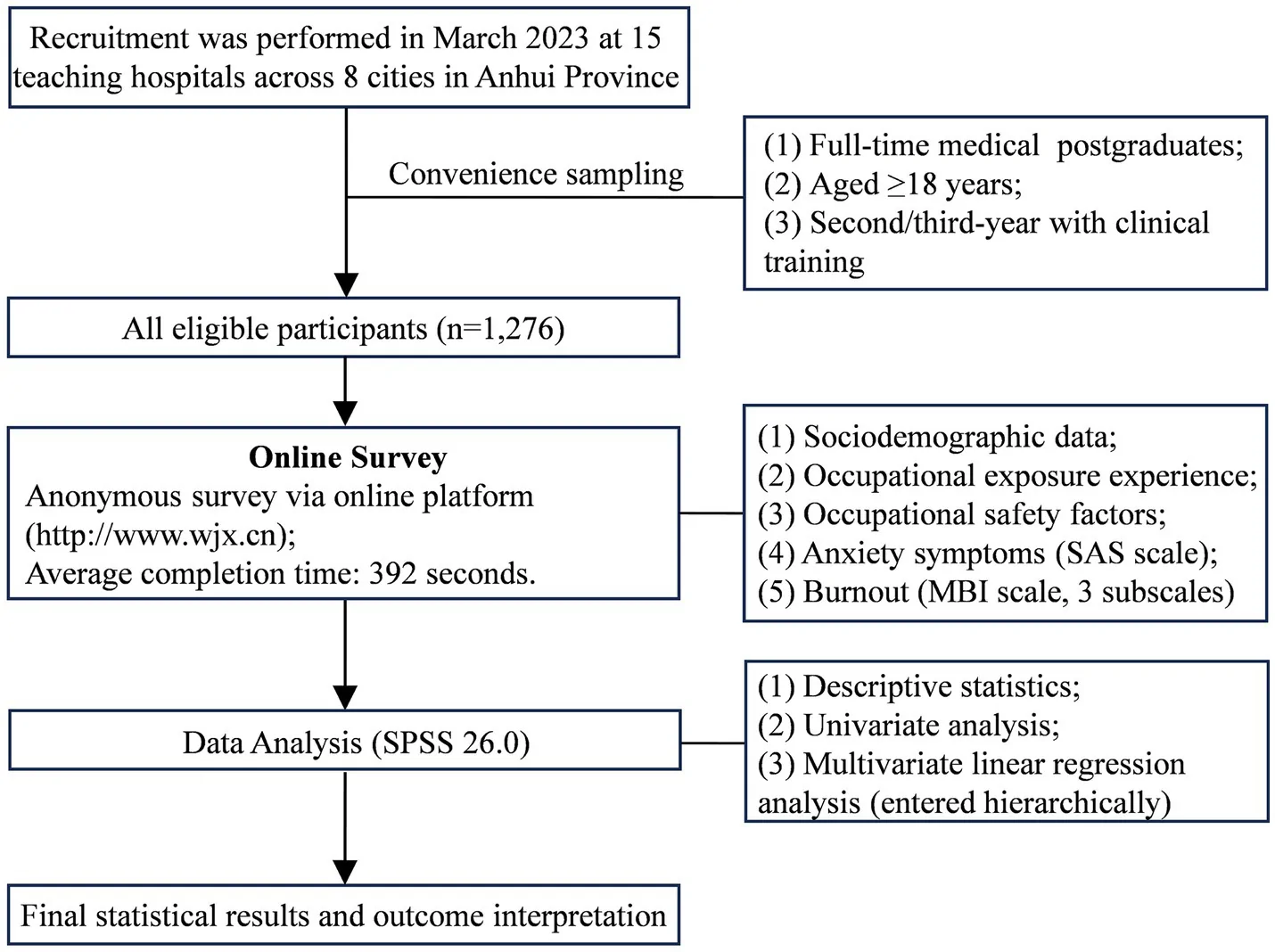
Flowchart of the study design and methodology.

### 2.2 Online survey procedure

The questionnaire survey was conducted using the Wenjuanxing online platform^1^, with questionnaires distributed online by the postgraduate management departments of the participating hospitals. Participants voluntarily completed the anonymous online questionnaire via the Wenjuanxing platform using mobile phones or computers, with no material incentives provided. Participants completed the questionnaire in a mean time of 392 s.

### 2.3 Survey instruments

A structured survey questionnaire was developed for data collection in this study, which was divided into the following sections.

#### 2.3.1 Sociodemographic characteristics

This section comprised eight items assessing participants’ sociodemographic characteristics, including age, gender, academic year (second year or third year), degree pursued (master or doctoral), degree type (professional or academic), major (internal medicine, intensive care/emergency medicine, surgery, pediatrics, obstetrics and gynecology, anesthesiology, and others), and formal medical institutional employment experience.

#### 2.3.2 Occupational exposure experience

This part included eight items to investigate the incidence and specific details of sharp injuries and splash exposures that may pose a risk of cross-infection during the 12-month period prior to this survey. A sharp injury was defined as a skin puncture or laceration caused by needles, scalpels, or other sharp objects during postgraduate training. Splash exposure was defined as direct contact of blood, body fluids, vomitus, or secretions with mucous membranes including the eyes, nasal cavity, and oral cavity, or with non-intact skin.

#### 2.3.3 Occupational safety factors

This section consisted of nine items evaluating key factors related to occupational safety, including attitudes toward occupational exposure, compliance with standard precautions, completion of occupational safety education at medical school and pre-training safety education at hospitals, as well as the availability and adequacy of personal protective equipment.

#### 2.3.4 Anxiety symptoms assessment

Anxiety symptoms were assessed using the Self-Rating Anxiety Scale (SAS), a self-administered measure evaluating the frequency of symptoms over the previous week (Zung, 1971). This instrument comprises 20 items that capture both affective and somatic manifestations of anxiety. For each item, participants indicated the frequency of symptom occurrence on a 4-point Likert scale, with response options ranging from 1 (a little of the time) to 4 (most of the time). A raw score was calculated by summing the item responses, and a standardized score was derived by multiplying the raw score by 1.25, with higher scores reflecting greater anxiety severity. In the current sample, the SAS demonstrated strong internal consistency, yielding a Cronbach’s *α* coefficient of 0.889.

#### 2.3.5 Burnout assessment

Burnout was evaluated using the Maslach Burnout Inventory (MBI), a standardized tool that measures three core dimensions: emotional exhaustion, depersonalization, and personal accomplishment (Maslach et al., 1996). This scale consists of 22 items rated on a 7-point frequency scale, in which 0 indicated “never” and 6 indicated “every day,” based on responses referring to the past week. To align the direction of all subscales, each personal accomplishment item was reverse-coded, and the sum of the reversed items represented reduced personal accomplishment. Higher scores indicated more severe burnout. Burnout was assessed across the three dimensions, with cutoff scores defined as emotional exhaustion ≥27, depersonalization ≥13, and personal accomplishment ≤31. Overall burnout severity was classified according to the number of dimensions meeting the cutoff: none for zero dimensions; mild for any one dimension, moderate for any two, and severe for all three. Cronbach’s *α* coefficients for these three subscales ranged from 0.876 to 0.921, demonstrating good to excellent internal consistency.

### 2.4 Data analysis

The collected data from Wenjuanxing were exported to Microsoft Excel format, and all statistical analyses were performed using SPSS 26.0 (SPSS Inc., Chicago, IL). Continuous variables were expressed as mean ± standard deviation (SD), while categorical variables were presented as frequencies and percentages. Independent-samples *t*-test was used for comparison between two groups, and one-way analysis of variance (ANOVA) for comparison among more than two groups.

Variables with statistically significant differences in univariate analysis were used as independent variables, and burnout was used as the dependent variable for multivariate linear regression analysis with the enter method. All variance inflation factor (VIF) values were <10, indicating no multicollinearity among independent variables. To clarify the impact of different variable types on burnout, variables were entered hierarchically: Model 1 included demographic characteristics; Model 2 added occupational exposure experience; Model 3 included occupational safety factors; and Model 4 incorporated anxiety symptoms. All statistical tests were two-tailed, and a *p* < 0.05 was considered statistically significant.

## 3 Results

### 3.1 Characteristics of study participants

This study enrolled 1,276 medical postgraduates with a mean age of 26.68 ± 2.65 years, and 57.29% of the participants were female (Table 1). Most participants were in their second academic year (52.12%), while 47.88% were in their third year. In terms of academic type, most participants were master’s degree candidates (92.95%), and professional postgraduates accounted for the majority (81.97%). Regarding major distribution, internal medicine represented the largest proportion (37.46%), followed by surgery (25.08%), with other majors including anesthesiology, obstetrics and gynecology, and pediatrics showing scattered distributions.

**Table 1:** Demographic characteristics of participating medical postgraduates (*n* = 1,276).

Item	Category/Statistic	*n*/mean	%/SD
Age (year)	Mean ± SD	26.68	2.65
Gender	Male	545	42.71
	Female	731	57.29
Grade	Second	665	52.12
	Third	611	47.88
Degree pursued	Master	1,186	92.95
	Doctor	90	7.05
Training type	Professional	1,046	81.97
	Academic	230	18.03
Specialty	Internal medicine	478	37.46
	Intensive care/emergency medicine	38	2.98
	Surgery	320	25.08
	Pediatrics	65	5.09
	Obstetrics and gynecology	62	4.86
	Anesthesiology	80	6.27
	Others*	233	18.26
Formal medical institutional employment experience	No	1,091	85.50
	Yes	185	14.50

*Others (*N* = 233) included medical technology disciplines (radiology, medical imaging, ultrasound, and laboratory medicine, *n* = 97), dentistry (*n* = 13), and remaining other specialties (*n* = 123).

### 3.2 Burnout among medical postgraduate*s*

The mean scores for emotional exhaustion, depersonalization, and reduced personal accomplishment were 23.35 ± 12.51, 8.77 ± 6.69, and 18.14 ± 11.19, respectively. Regarding burnout severity, 30.02% of participants had no burnout, 32.68% had mild burnout, 20.22% had moderate burnout, and 17.07% had severe burnout.

Univariate analyses of three dimensions of the MBI are summarized in Table 2.

**Table 2:** Univariate analysis of burnout dimensions among medical postgraduates.

Variable	Category/Statistic	*n*	Emotional exhaustion	Depersonalization	Reduced personal accomplishment
Age	*r*		−0.099	−0.111	−0.055
	*P*		<0.001	<0.001	0.050
Gender	Male	545	22.88 ± 12.68	8.92 ± 6.96	18.57 ± 12.03
	Female	731	23.71 ± 12.38	8.66 ± 6.48	17.81 ± 10.53
	*t*		−1.181	0.676	1.186
	*P*		0.238	0.499	0.236
Grade	Second	665	23.76 ± 12.63	9.04 ± 6.88	18.59 ± 11.36
	Third	611	22.92 ± 12.37	8.48 ± 6.46	17.64 ± 10.99
	*t*		1.198	1.503	1.512
	*P*		0.231	0.133	0.131
Degree pursued	Master	1,186	23.81 ± 12.54	9.05 ± 6.70	18.33 ± 11.10
	Doctor	90	17.41 ± 10.52	5.12 ± 5.28	15.62 ± 12.12
	*t*		5.480	6.663	2.213
	*P*		<0.001	<0.001	0.027
Training type	Professional	1,046	24.13 ± 12.40	9.12 ± 6.74	18.52 ± 11.05
	Academic	230	19.82 ± 12.43	7.17 ± 6.18	16.41 ± 11.70
	*t*		4.771	4.026	2.590
	*P*		<0.001	<0.001	0.010
Specialty	Internal medicine	478	23.44 ± 12.58	8.69 ± 6.64	18.56 ± 11.47
	Intensive care/emergency medicine	38	20.37 ± 11.72	6.05 ± 5.63	18.71 ± 12.27
	Surgery	320	23.53 ± 12.03	9.22 ± 6.70	17.91 ± 11.52
	Pediatrics	65	21.28 ± 12.56	7.43 ± 6.13	15.51 ± 10.12
	Obstetrics and gynecology	62	24.71 ± 13.22	9.29 ± 6.98	18.03 ± 11.15
	Anesthesiology	80	30.93 ± 12.72	11.88 ± 7.49	18.74 ± 10.06
	Others	233	21.05 ± 11.85	7.93 ± 6.33	18.05 ± 10.66
	*F*		7.200	5.395	0.790
	*P*		<0.001	<0.001	0.578
Formal medical institutional employment experience	No	1,091	23.76 ± 12.35	9.02 ± 6.63	18.29 ± 11.03
	Yes	185	20.96 ± 13.20	7.32 ± 6.85	17.22 ± 12.12
	*t*		2.821	3.207	1.208
	*P*		0.005	0.001	0.227
Sharp injuries in the past year	No	748	21.27 ± 11.98	7.75 ± 6.41	17.19 ± 11.24
	Yes	528	26.30 ± 12.66	10.21 ± 6.81	19.47 ± 11.00
	*t*		−7.211	−6.584	−3.593
	*P*		<0.001	<0.001	<0.001
Splash exposures in the past year	No	912	21.97 ± 11.95	8.04 ± 6.51	17.48 ± 11.07
	Yes	364	26.81 ± 13.20	10.60 ± 6.79	19.78 ± 11.36
	*t*		−6.071	−6.252	−3.334
	*P*		<0.001	<0.001	0.001
Concern regarding occupational exposure	No	378	21.16 ± 12.56	7.78 ± 6.69	16.97 ± 11.66
	Yes	898	24.28 ± 12.38	9.19 ± 6.65	18.63 ± 10.96
	*t*		−4.098	−3.458	−2.421
	*P*		<0.001	0.001	0.016
Comply with clinical standard operating procedures	No	717	25.24 ± 12.07	9.85 ± 6.60	19.26 ± 10.40
	Yes	559	20.94 ± 12.66	7.38 ± 6.54	16.69 ± 11.99
	*t*		6.183	6.660	4.015
	*P*		<0.001	<0.001	<0.001
University occupational safety education	No	611	24.73 ± 13.05	9.48 ± 7.09	19.67 ± 11.55
	Yes	665	22.09 ± 11.86	8.12 ± 6.23	16.72 ± 10.68
	*t*		3.775	3.626	4.741
	*P*		<0.001	<0.001	<0.001
Hospital pre-training safety education	No	619	24.94 ± 13.01	9.71 ± 7.10	19.07 ± 11.10
	Yes	657	21.86 ± 11.83	7.89 ± 6.15	17.25 ± 11.22
	*t*		4.425	4.871	2.906
	*P*		<0.001	<0.001	0.004
Adequate access to personal protective equipment	No	1,145	23.67 ± 12.52	8.96 ± 6.72	18.29 ± 11.11
	Yes	131	20.56 ± 12.05	7.16 ± 6.18	16.76 ± 11.86
	*t*		2.701	2.920	1.490
	*P*		0.007	0.004	0.136
Anxiety symptoms	*r*		0.459	0.493	0.480
	*P*		<0.001	<0.001	<0.001

### 3.3 Factors associated with emotional exhaustion among medical postgraduates

In Model 1 (Table 3), demographic characteristics accounted for 6.1% of the total variance in emotional exhaustion among medical postgraduates. After incorporating variables related to occupational exposure experience, Model 2 explained 8.1% of the total variance. With the further addition of occupational safety factors, the explanatory power of Model 3 increased to 12.2%. Finally, Model 4, which included anxiety symptoms, explained 26.1% of the total variance in emotional exhaustion.

**Table 3:** Multivariate analysis of emotional exhaustion among medical postgraduates.

Variable	Model 1		Model 2		Model 3		Model 4	
	*β*	*P*	*β*	*P*	*β*	*P*	*β*	*P*
Constant	38.626	<0.001	28.320	<0.001	27.173	<0.001	13.915	0.001
Age	−0.133	0.422	−0.085	0.603	−0.034	0.835	−0.245	0.099
Academic (vs. professional)	−4.242	<0.001	−2.929	0.002	−3.042	0.001	−2.161	0.010
Doctor (vs. master)	−5.741	<0.001	−4.526	0.002	−4.053	0.005	−2.439	0.068
Intensive care/emergency medicine (vs. internal medicine)	−2.722	0.185	−3.327	0.102	−2.987	0.135	−2.661	0.147
Surgery (vs. internal medicine)	0.840	0.343	−0.275	0.760	0.150	0.865	0.927	0.254
Pediatrics (vs. internal medicine)	−2.522	0.116	−2.313	0.146	−2.069	0.184	−0.922	0.519
Obstetrics and gynecology (vs. internal medicine)	1.815	0.272	1.116	0.496	0.958	0.551	0.813	0.581
Anesthesiology (vs. internal medicine)	7.147	<0.001	5.405	<0.001	5.608	<0.001	4.943	<0.001
Others (vs. internal medicine)	−2.023	0.037	−1.897	0.049	−1.335	0.157	−0.807	0.352
Formal medical institutional employment experience	−0.644	0.579	−0.925	0.421	−0.816	0.468	−0.077	0.940
Sharp injuries in the past year			2.681	<0.001	2.219	0.003	1.434	0.038
Splash exposures in the past year			2.433	0.003	2.507	0.002	0.716	0.345
Concern regarding occupational exposure					3.504	<0.001	1.991	0.004
Comply with clinical standard operating procedures					−2.985	<0.001	−2.085	0.001
University occupational safety education					−1.367	0.108	−0.244	0.756
Hospital pre-training safety education					−1.318	0.126	−1.526	0.054
Adequate access to personal protective equipment					−1.778	0.105	−1.690	0.093
Anxiety symptoms							0.464	<0.001
*R* ^2^	0.0683		0.0895		0.1334		0.2717	
Adjusted *R*^2^	0.0609		0.0809		0.1217		0.2612	

The fully adjusted model (Model 4) revealed that being an academic postgraduate (*β* = −2.161, *p* = 0.010) and strict compliance with standard operating procedures (*β* = −2.085, *p* = 0.001) were independently associated with lower levels of emotional exhaustion. Conversely, majoring in anesthesiology (*β* = 4.943, *p* < 0.001), a history of sharp injury within the past year (*β* = 1.434, *p* = 0.038), concern about occupational exposure (*β* = 1.991, *p* = 0.004), and anxiety symptoms (*β* = 0.464, *p* < 0.001) were significantly correlated with higher levels of emotional exhaustion.

### 3.4 Factors associated with depersonalization among medical postgraduates

In Model 1 (Table 4), demographic characteristics accounted for 5.6% of the total variance in depersonalization among medical postgraduates. After incorporating variables related to occupational exposure experience, Model 2 explained 7.4% of the total variance. With the further addition of occupational safety factors, the explanatory power of Model 3 increased to 11.7%. Finally, Model 4, which included anxiety symptoms, explained 29.2% of the total variance in depersonalization.

**Table 4:** Multivariate analysis of depersonalization among medical postgraduates.

Variable	Model 1		Model 2		Model 3		Model 4	
	*β*	*P*	*β*	*P*	*β*	*P*	*β*	*P*
Constant	17.370	<0.001	12.174	<0.001	12.131	<0.001	4.190	0.045
Age	−0.076	0.393	−0.052	0.554	−0.022	0.798	−0.149	0.056
Academic (vs. professional)	−1.987	<0.001	−1.321	0.008	−1.424	0.004	−0.897	0.041
Doctor (vs. master)	−3.649	<0.001	−3.040	<0.001	−2.846	<0.001	−1.880	0.007
Intensive care/emergency medicine (vs. internal medicine)	−2.415	0.028	−2.715	0.013	−2.484	0.021	−2.288	0.017
Surgery (vs. internal medicine)	0.946	0.047	0.383	0.429	0.616	0.194	1.081	0.011
Pediatrics (vs. internal medicine)	−1.470	0.088	−1.376	0.107	−1.282	0.124	−0.595	0.426
Obstetrics and gynecology (vs. internal medicine)	0.794	0.370	0.432	0.623	0.300	0.727	0.213	0.782
Anesthesiology (vs. internal medicine)	2.988	<0.001	2.126	0.008	2.211	0.005	1.813	0.010
Others (vs. internal medicine)	−0.580	0.265	−0.512	0.321	−0.208	0.681	0.109	0.810
Formal medical institutional employment experience	−0.463	0.456	−0.614	0.319	−0.561	0.353	−0.118	0.827
Sharp injuries in the past year			1.212	0.003	0.929	0.021	0.459	0.206
Splash exposures in the past year			1.390	0.002	1.338	0.002	0.264	0.505
Concern regarding occupational exposure					1.595	<0.001	0.689	0.055
Comply with clinical standard operating procedures					−1.742	0.000	−1.202	<0.001
University occupational safety education					−0.515	0.258	0.157	0.701
Hospital pre-training safety education					−1.049	0.023	−1.174	0.005
Adequate access to personal protective equipment					−1.113	0.058	−1.061	0.044
Anxiety symptoms							0.278	<0.001
*R* ^2^	0.0638		0.0829		0.1287		0.3023	
Adjusted *R*^2^	0.0564		0.0742		0.1169		0.2923	

The fully adjusted model (Model 4) revealed that being an academic postgraduate (*β* = −0.897, *p* = 0.041), pursuing a doctoral degree (*β* = −1.880, *p* = 0.007), majoring in intensive care/emergency medicine (*β* = −2.288, *p* = 0.017), strict compliance with standard operating procedures (*β* = −1.202, *p* < 0.001), hospital pre-training safety education (*β* = −1.174, *p* = 0.005), and adequate access to personal protective equipment (*β* = −1.061, *p* = 0.044) were independently associated with lower levels of depersonalization. Conversely, majoring in surgery (*β* = 1.081, *p* = 0.011), majoring in anesthesiology (*β* = 1.813, *p* = 0.010), and anxiety symptoms (*β* = 0.278, *p* < 0.001) were significantly correlated with higher levels of depersonalization.

### 3.5 Factors associated with reduced personal accomplishment among medical postgraduates

In Model 1 (Table 5), demographic characteristics accounted for 0.6% of the total variance in reduced personal accomplishment among medical postgraduates. After incorporating variables related to occupational exposure experience, Model 2 explained 1.3% of the total variance. With the further addition of occupational safety factors, the explanatory power of Model 3 increased to 4.0%. Finally, Model 4, which included anxiety symptoms, explained 23.6% of the total variance in reduced personal accomplishment.

**Table 5:** Multivariate analysis of reduced personal accomplishment among medical postgraduates.

Variable	Model 1		Model 2		Model 3		Model 4	
	*β*	*P*	*β*	*P*	*β*	*P*	*β*	*P*
Constant	26.628	<0.001	20.671	<0.001	21.562	<0.001	7.521	0.039
Age	−0.117	0.445	−0.089	0.558	−0.081	0.591	−0.305	0.024
Academic (vs. professional)	−2.132	0.010	−1.372	0.110	−1.479	0.083	−0.546	0.475
Doctor (vs. master)	−2.382	0.078	−1.682	0.217	−1.468	0.279	0.241	0.842
Intensive care/emergency medicine (vs. internal medicine)	0.344	0.855	−0.003	0.999	−0.207	0.912	0.138	0.934
Surgery (vs. internal medicine)	−0.270	0.741	−0.915	0.274	−0.740	0.371	0.083	0.910
Pediatrics (vs. internal medicine)	−3.192	0.031	−3.077	0.037	−2.982	0.041	−1.767	0.175
Obstetrics and gynecology (vs. internal medicine)	−0.236	0.876	−0.645	0.671	−0.820	0.586	−0.973	0.468
Anesthesiology (vs. internal medicine)	0.025	0.985	−0.974	0.480	−0.852	0.532	−1.556	0.201
Others (vs. internal medicine)	−0.334	0.709	−0.259	0.771	0.085	0.923	0.645	0.414
Formal medical institutional employment experience	−0.020	0.985	−0.187	0.861	−0.050	0.962	0.733	0.436
Sharp injuries in the past year			1.478	0.037	1.156	0.101	0.325	0.607
Splash exposures in the past year			1.490	0.050	1.545	0.043	−0.353	0.609
Concern regarding occupational exposure					1.696	0.014	0.094	0.880
Comply with clinical standard operating procedures					−1.959	0.002	−1.005	0.081
University occupational safety education					−3.076	<0.001	−1.886	0.008
Hospital pre-training safety education					0.519	0.519	0.299	0.677
Adequate access to personal protective equipment					−0.846	0.409	−0.753	0.410
Anxiety symptoms							0.492	<0.001
*R* ^2^	0.0139		0.0227		0.0527		0.2464	
Adjusted *R*^2^	0.0061		0.0134		0.0399		0.2356	

The fully adjusted model (Model 4) revealed that advanced age (*β* = −0.305, *p* = 0.024) and university occupational safety education (*β* = −1.886, *p* = 0.008) were independently associated with lower levels of reduced personal accomplishment. Conversely, anxiety symptoms (*β* = 0.492, *p* < 0.001) were significantly correlated with higher levels of reduced personal accomplishment.

## 4 Discussion

The present study investigated the prevalence of burnout and its associated factors among 1,276 medical postgraduates. The results revealed that 69.97% of participants had mild to severe burnout, among whom 17.07% had severe burnout. This indicates that burnout is a prevalent and concerning issue among medical postgraduates, consistent with the global reports of elevated burnout risk in high-pressure medical training environments (Liu et al., 2023; Prentice et al., 2020; Priyam et al., 2024). Multiple hierarchical multivariate regression revealed a gradual improvement in the model’s explanatory variance for burnout. Specifically, sociodemographic characteristics, occupational exposure experiences, occupational safety factors, and anxiety symptoms collectively accounted for burnout vulnerability, with anxiety demonstrating the strongest explanatory effect.

Consistent with prior research in medical students (Gilbey et al., 2023), this study found that age was significantly negatively associated with burnout, suggesting older medical postgraduates may possess greater psychological resilience and coping strategies to buffer training pressure and reduce burnout levels. Professional postgraduates reported significantly higher burnout than academic counterparts, which may stem from their heavier clinical workload and greater clinical-risk exposure leading to increased emotional and physical exhaustion (Prentice et al., 2020). Previously published research has documented that while higher degrees serve as protective factors against burnout among non-physicians, a higher medical degree becomes a risk factor among physicians, revealing a notable disparity across professions (Shanafelt et al., 2012). However, inconsistent with the situation among physicians, medical master’s students in this study exhibited significantly higher depersonalization than doctoral students. This discrepancy may partly reflect Chinese medical education context, where most medical master’s students undertake both standardized clinical rotations and research tasks, whereas doctoral students focus primarily on scientific research. Consistent with previous evidence among physicians (Sun et al., 2025), the present study observed significant differences in burnout risk across postgraduate students from different clinical specialties, with anesthesiology emerging as one of the particular specialties of concern. This observation supports the view that specialty-specific occupational challenges contribute to stress and subsequent burnout syndrome (Panditrao and Panditrao, 2024). Accordingly, greater attention should be directed toward high-burnout specialties.

Occupational exposure experience and safety factors also play an important role in burnout in medical postgraduates. Occupational exposure represents a common and often unavoidable risk faced by medical postgraduates and other medical workers (Hambridge, 2023; Kaddour et al., 2026; Praveen Kumar et al., 2019). This study found that a history of sharp injury in the past year and concern about occupational exposure were significantly associated with higher emotional exhaustion, in line with prior work showing occupational exposure promotes psychological stress and further induces burnout among medical workers (Ercömert et al., 2025; Hambridge et al., 2022). Notably, this relationship may be bidirectional. Burnout subdimensions have been linked to occupational accidents among resident physicians (Ahola et al., 2013; Ercömert et al., 2025). Burnout-related physical and mental impairment can deplete personal resources and further raise the risk of sharp injuries and other occupational incidents (Ahola et al., 2013). In addition, strict compliance with standard operating procedures, university- and hospital-provided safety education, and adequate access to personal protective equipment were associated with lower burnout subdimensions. This provides a new insight that improving occupational protection conditions and strengthening safety training can effectively alleviate burnout in medical postgraduates. On one hand, better occupational protection and safety training reduce the occurrence of occupational exposures (Cheetham et al., 2021), a well-established contributor to burnout. On the other hand, they may enhance postgraduates’ confidence and alleviate safety-related concerns, thereby decreasing burnout risk regardless of actual exposure events. These findings highlight the value of safety-focused interventions for burnout prevention in this population.

Notably, this study confirmed that anxiety symptoms are the strongest predictor of burnout, consistent with previous studies indicating a close association between mental health conditions, such as anxiety, and burnout among medical students and trainees (Pokhrel et al., 2020; Tang et al., 2023). One previous study demonstrated that all three dimensions of burnout are significantly correlated with anxiety in medical workers (Tang et al., 2023), while another study also identified anxiety as a positive predictor of burnout among medical students and residents (Pokhrel et al., 2020). The inclusion of anxiety symptoms significantly improved the explanatory power of the prediction model, highlighting that anxiety is the most critical factor influencing burnout among medical postgraduates. This underscores the necessity of embedding mental health interventions into strategies for mitigating burnout among medical postgraduates, particularly through the early identification and management of anxiety symptoms.

Several limitations of this study should be acknowledged. First, this study adopted a cross-sectional design, which can identify associations but limits causal inference. For instance, although anxiety symptoms are significantly associated with burnout, whether anxiety triggers burnout or burnout aggravates anxiety warrants further longitudinal studies. Second, this study relied on self-reported questionnaires to assess burnout and its associated factors, which may introduce response biases. Notably, burnout assessed via MBI may not fully capturing the theoretical burnout syndrome itself. Third, we could not collect data on several unmeasured confounding variables, including weekly duty hours, monthly night shifts, stipend satisfaction, and patient-to-trainee ratios, which may exert potential influences on the observed associations. Fourth, despite the relatively large sample size, we adopted convenience sampling, which may introduce potential selection bias. Additionally, this study recruited medical postgraduates from Anhui Province. The local residency training system follows national standard requirements, though regional variation in clinical workload persists. These findings are broadly representative of Chinese medical postgraduates under standardized training, yet regional heterogeneity should be considered when extrapolating findings.

Findings from this study have important practical implications for clinical management and medical education optimization. First, targeted interventions should consider subgroup characteristics. For professional and master’s students, training arrangements should balance clinical rotation with academic research to reduce redundant workload. For high-risk specialties such as anesthesiology, psychological support and rationalized work schedules are needed to relieve occupational pressure and burnout risk. Second, medical institutions and universities should ensure adequate personal protective equipment and systematically strengthen safety pre-training, thereby improving self-protection awareness and alleviating psychological stress caused by concerns over occupational exposure. Third, given that anxiety is the most powerful predictor of burnout, regular psychological assessments, professional counseling, and stress coping training should be integrated into routine management to enable early identification and mitigation of anxiety and subsequent burnout among medical postgraduates.

## 5 Conclusion

In conclusion, the majority of medical postgraduates experience burnout with varying severity, and its occurrence is affected by multiple factors, including sociodemographic characteristics, occupational exposures, occupational safety factors, and anxiety symptoms. These findings provide valuable evidence for recognizing the overall status and associated factors of burnout among medical postgraduates. Targeted interventions concerning medical education management, occupational protection, and mental health support are urgently required to reduce burnout, optimize postgraduates’ mental health status and facilitate the high-quality development of medical education. Future studies should adopt larger, multi-region samples, and longitudinal designs to improve the external validity of the findings.

## Data Availability

The raw data supporting the conclusions of this article will be made available by the authors, without undue reservation.

## Footnotes

1
http://www.wjx.cn
